# Editorial

**Published:** 1871-12

**Authors:** 


### EDITORIAL.

Our French Exchanges.—The Paris medical journals were interrupted for a time during the Franco-Prussian war. The missing numbers, however, have all been replaced, and the issues resumed as usual.

The subject of *purulent infection* had been under discussion at the Academy of Medicine for several months before the war, and has since been resumed. The *Gazette Mêdicale de Paris* in an editorial comment on the discussion says, "The discussion in regard to purulent infection which has been continued at the Academy of Medicine for a number of months is certainly one of the most interesting ever recorded in the annals of that illustrious society. It would be difficult to find a subject offering an equally vast field for the collection of facts and ideas. The highest conceptions of general pathology have been called forth by it, as well as clinical observations the most simple, the most empirical.

With certain orators we have been transported for a time into the highest regions of metaphysics, while in following others we do not leave the solid ground of practical journalism.

All this comes, however, within the limits of the subject, for in medicine the theory and the practice are inseperable. The first illuminates the second; the one confirms or demonstrates the other. In the midst of all the doctrines, however, the only truth is found by careful observation of well demonstrated facts. It is comparatively easy to form an idea à priori, to conceive an hypothesis, or to elucidate a theory, but it is a much more difficult task to make careful observations and from them to establish true reports, real and exact."

M. Verneuil, in the course of his discussion, said: "In considering the subject of purulent infection there are three points to be noted, the person wounded, the wound, and the media or atmosphere surrounding the patient. The wisest course is, without doubt, to give equal consideration to each of the three, but, according to the doctrine which we profess we are apt to accord the preëminence to one or the other. For instance, M. Chauffard, with his ideas fixed upon the spontaneous growth of organisms through the activity of plastic life, sees before all else the wound; M. Alphonse Guérin, with his "theory miamatique," on the other hand, has his attention entirely pre-occupied with the *atmosphere* surrounding his patient, and the *poisons brought by it* to the wound. Just here, without any doubt, arises the difference in the practice of the one and the other confere."

---

Rush Medical College. Correction.—In the October number of the Examiner, in alluding to the effects of the recent great fire on the medical colleges of this city, we stated that the Faculty of the Rush Medical College resumed their regular course of instruction, in the lecture room of the County Hospital, in about two weeks. We made the statement without any positive data, and we have since been informed by the Secretary, that "the lectures of the Faculty of the Rush Medical College, were interrupted but *four days* by the fire; since which time the course has been given regularly." We take pleasure in making the correction.

---

THE PRESENT AND THE NEXT VOLUME.—We had intended to issue each number of the present volume of the EXAMINER separately. But the unexpected delay on the part of our old printers, in getting new presses since the fire, coupled with our desire to have the new volume commence by the issue of the January number promptly during the first week in the month, has caused us to put the November and December numbers together, thereby completing the present volume with this issue. The new volume, commencing with the January number, will be published regularly, in good style, and with much more complete arrangements for making every department of it more valuable to the practitioner than heretofore. We cordially thank our patrons for past favors, and hope they will find it for their interests to continue the same in the future. A few of our subscribers are still in arrears. We hope they will appreciate the importance of early remittance.

---

CHICAGO MEDICAL SOCIETY.—This society resumed its regular meetings on the first and third Monday evenings of each month, soon after the fire. Owing to the absence of any central place of meeting, the number in attendance has usually been small. This will be remedied in a few months, when the society will probably become more prosperous than at any former period.

---

ILLINOIS STATE MICROSCOPICAL SOCIETY.—The regular scientific meetings of this society, which had been interrupted since the fire, were resumed on Friday evening last, December 8th, with a fair proportion of the active members in attendance.

Prof. H. H. Babcock read an interesting paper on "the Diatoms of Chicago Hydrant Water." After recounting the principal species usually found in our hydrant water, he stated that, since the completion of the canal improvement, changing the current of the Chicago river so as to cause it to flow from the lake instead of into it, the diatoms had very much diminished in numbers, some of the species having almost entirely disappeared, and that the amount of other organic materials found in the water was also very much less than previously. It was evident that the change in the current of the river had very materially increased the purity of the hydrant water, and this improvement in the water has probably been of considerable benefit to the sanitary condition of the city. It would appear that the foul impure water from the river as it flowed into the lake must have mingled with and contaminated, to a certain extent, the lake water even as far out as the crib, two miles from shore, which had been supposed to be beyond the influence of the river water. There was, however, another theory by which he thought the increased purity of the water might, perhaps, be accounted for. The existence of a tolerably constant, uniform current flowing through the lake from north to south along parallel with, and at a short distance from, the shore has been at least partially proven. While on a visit to Mackinac at the north end of Lake Michigan last summer he had found in specimens of the lake water taken from near the mouth of Carp river diatoms of the same species as those observed here. This fact suggested the idea that the crib might have been in the course of this current and the diatoms and other organic impurities found in the hydrant water have thus been derived from it. The change in the course of the river however, having drawn the current in nearer to the shore left the crib outside, beyond the reach of its influence, in the pure water. Whichever of the theories may be true, or whether either of them is correct or not, is of little consequence beside of the fact that our city is now being supplied with water almost absolutely free from organic impurities.

In the concluding paragraph of the paper it was, however, stated that for the past few weeks the current in the river had not been as rapid and as a consequence the hydrant water had begun again to approximate its old standard of impurity.

At the conclusion of the reading of this paper Mr. H. W. Fuller made a few remarks in regard to the proper care of the microscope and microscopic apparatus ; the discussion of the subject being continued in an informal manner by Prof. Babcock, Dr. Johnson and others. The society then adjourned its formal meeting, the members remaining however for a few minutes to examine slides, instruments, etc. Mr. H. M. Thompson had on exhibition a very handsome and complete new microscope, one of Smith, Beck & Beck's finest instruments which he had just imported.

---

SECRETARY'S OFFICE OF THE ILLINOIS STATE MEDICAL SOCIETY, CHICAGO, NOV. 1, 1871.—*To the members of the Illinois State Medical Society,* GENTLEMEN,—The transactions of this Society were nearly ready for distribution, but yet in the hands of the publishers at the time of our great fire. The publishers lost everything, and the State Medical Society their transactions for this year. Nothing was saved except a copy of the proceedings which was in our hands, and a few of the articles and reports that had been published in the medical journals and others in pamphlet form by their authors.

We shall at once put these into type again and obtain, if possible, duplicate copies of all the unpublished reports from their authors.

We ask the indulgence of the Society for our seeming tardiness in issuing the transactions, but many circumstances which seemed beyond our control, conspired to delay the issue.

We have made direct application to the authors of all unpublished papers, and hope to be able to issue nearly, if not quite, a complete copy within the next thirty days.

In behalf of the Committee on Publication.

T. D. FITCH, M. D.,
*Permanent Secretary.*

### Mortality for October, commencing with the 8th.

(In the first seven days there were 88 deaths.)

| | | | | | |
|---|---|---|---|---|---|
| Accident by bridge | 1 | Cystitis | 1 | Liver disease of | 1 |
| " " burns | 96 | Debility, general | 6 | " and bowels, do | 1 |
| " " wagon | 1 | Diabetes | 1 | " hypertophy of | 1 |
| " " drowning | 8 | Diarrhœa | 8 | " ulceration of | 1 |
| " " concussion of | | " chronic | 7 | Lungs, abscess of | 1 |
| brain | 1 | Diphtheria | 9 | Manslaughter | 6 |
| Accident by falling walls, | 5 | Dropsy, general | 4 | Meningitis | 7 |
| " " fall | 2 | Dysentery | 7 | " cerebro-spinal, | 3 |
| " " kick by horse, | 1 | " chronic | 2 | " tubercular | 2 |
| " " by railroad | 1 | Enteritis | 11 | Menth canker sore | 6 |
| Abcess lumbar | 1 | Exhaustion | 1 | Metritis puerperal | 6 |
| " sloughing of | 1 | Epilepsy | 1 | Old age | 4 |
| Anæmia | 1 | Exposure | 5 | Paralysis | 9 |
| Aneurism of aorta | 1 | Erysipelas | 3 | Pneumonia | 22 |
| Alcoholic poisoning | 1 | Fever, intermittent | 3 | " typhoid | 2 |
| Apoplexy | 2 | " puerperal | 4 | Rheumatism | 1 |
| Asphyxia | 1 | " remittent | 1 | Scrofula | 1 |
| Asthma | 1 | " scarlet | 15 | Small pox | 1 |
| Bowels, intussusseption | | " " malignant | 1 | Spina bifida | 1 |
| of | 1 | " typhoid | 18 | Stomach, hemmorage of | 1 |
| " and stomach, con- | | Fright | 1 | " ulceration of | 1 |
| gestion of | 1 | Gangrene | 1 | Suicide by shooting | 1 |
| Brain, congestion of | 6 | Gastritis | 2 | " by drowning | 1 |
| " inflammation of | 5 | Gastro-enteritis | 1 | Tabes mesenterica | 13 |
| Bronchitis | 6 | Heart, disease of | 3 | Teething | 3 |
| " chronic | 1 | " and liver, do | 1 | Whooping cough | 2 |
| Cancer of breast | 1 | " hypertrophy of | 2 | Unknown | 17 |
| " face | 1 | Hemiplegia | 1 | | — |
| " liver | 1 | Hepatitis | 1 | Total | 542 |
| " uterus | 1 | Hydrocephalus | 9 | | |
| Carbuncle gangrene | 1 | Inanition | 7 | | |
| Cholera infantum | 21 | Kidneys, Bright's dis- | | | |
| Cholera morbus | 1 | ease of | 3 | | |
| Consumption | 35 | Kidneys, inflammation of, | 1 | Premature births | 11 |
| Convulsions | 57 | Laryngitis | 1 | Still do | 26 |
| " puerperal | 1 | Lead colic | 1 | | — |
| Croup | 22 | Liver, congestion of | 1 | Total | 37 |

### AGES.

| | | | | | |
|---|---|---|---|---|---|
| Under 1 | 158 | 70 to 80 | 10 | Colored | 4 |
| 1 to 2 | 47 | 80 to 90 | 2 | White | 538 |
| 2 to 3 | 29 | Unknown | 108 | | — |
| 3 to 4 | 16 | | — | Total | 542 |
| 4 to 5 | 10 | Total | 542 | | |
| 5 to 10 | 15 | | | | |
| 10 to 20 | 15 | | | Married | 126 |
| 20 to 30 | 32 | Males | 257 | Single | 331 |
| 30 to 40 | 30 | Females | 200 | Unknown | 85 |
| 40 to 30 | 36 | Unknown | 85 | | — |
| 50 to 60 | 21 | | — | Total | 542 |
| 60 to 70 | 13 | Total | 542 | | |

### MORTALITY BY WARDS.

| Wards. | No. deaths. | Pop'n 1871. | Per c't. |
|---|---|---|---|
| 1 | ... | 8,103 | |
| 2 | ... | 13,449 | |
| 3 | 23 | 17,934 | 779 |
| 4 | 5 | 14,022 | 2,804 |
| 5 | 17 | 14,991 | 882 |
| 6 | 28 | 22,018 | 818 |
| 7 | 27 | 15,590 | 577 |
| 8 | 37 | 25,420 | 687 |
| 9 | 53 | 50,778 | 581 |
| 10 | 23 | 17,292 | 752 |
| 11 | 25 | 16,212 | 648 |
| 12 | 21 | 15,018 | 715 |
| 13 | 8 | 9,740 | 1,217 |
| 14 | 20 | 9,339 | 467 |
| 15 | 66 | 25,707 | 289 |
| 16 | 12 | 16,380 | 1,365 |
| 17 | 4 | 18,814 | 4,702 |
| 18 | 3 | 18,805 | 6,268 |
| 19 | ... | 9,236 | ...... |
| 20 | 2 | 14,522 | ...... |

RECAPITULATION.

| | |
|---|---|
| Unknown wards | 12 |
| Accidents | 116 |
| County hospital | 8 |
| Alexian Brothers | 1 |
| Carpenter School | 1 |
| Foundlings' Home | 8 |
| Half Orphan Asylum | 1 |
| Home for Friendless | 1 |
| Manslaughter | 6 |
| Police Station | 1 |
| Mercy Hospital | 6 |
| Suicides | 2 |
| Washingtonian House | 1 |
| Scotch Chnrch | 1 |
| Barracks | 2 |
| Burlington Depot | 1 |
| | — |
| Total | 542 |

NATIVITY.

| | |
|---|---|
| Bohemia | 6 |
| Canada | 5 |
| Chicago, native | 74 |
| " foreign | 169 |
| U. S., other parts | 50 |
| Denmark | 1 |
| England | 14 |
| France | 1 |
| Germany | 38 |
| Holland | 2 |
| Ireland | 40 |
| Norway | 10 |
| Scotland | 11 |
| Sweden | 6 |
| Unknown | 116 |
| Total | 542 |
| Deaths daily | 22½ |

This is necessarily the most incomplete report that I have ever presented. Although the number of deaths is about the same as last year, the causes are greatly changed. In this number there are 116 deaths from accidental causes, and of these there were 101 the result of the fire, either by burns or by falling walls. The total number of lives lost by the fire has not yet been ascertained, and I am satisfied that the remains of some will never be found, while others will be discovered when the *debris* of the buildings is removed. Judging from the remains that have already been found, I am inclined to the opinion that the loss of life will not exceed 200 by the fire. In this connection I would recommend that this Board keep a register of the missing. This has already been done partially, but owing to the multiplicity of cases, has been neglected for some time.

The general health of the city during this month has been unusually good, and from the foregoing report it will be seen that the causes of death were very few, independent of those directly the result of the fire. In October, 1868, there were 449 deaths, in 1869 601, and in 1870 about 575.

JOHN H. RANCH, M. D.

### MORTALITY FOR NOVEMBER 1871.

| | |
|---|---|
| Accident, burns | 1 |
| " drowning | 1 |
| " by fall | 1 |
| " " of wall | 2 |
| " gun-shot wound, | 1 |
| " gasoline | 1 |
| " run over by omnibus | 1 |
| " run over, horse cars | 1 |
| " run over by railroad cars | 4 |
| " scalding | 2 |
| Amputation for injury to foot | 1 |
| Aneurism of aorta | 1 |
| Apoplexy | 2 |
| Asthma | 2 |
| Atectasis pulmonary | 1 |
| Bowels, hemorrage of | 2 |
| " ulceration of | 1 |
| Brain, congestion of | 7 |
| " inflammation of | 18 |
| " softening of | 1 |
| Bronchitis | 4 |
| " capillary | 1 |
| Cancer of liver | 3 |
| " stomach | 1 |
| " uterus | 1 |
| Calculus | 1 |
| Cholera infantum | 7 |
| Colitis | 1 |
| Consumption | 32 |
| Convulsions | 50 |
| Croup | 16 |
| " membranous | 5 |
| Cyanosis | 2 |
| Cyananche trochealis | 1 |
| Debility, general | 7 |
| Delirium tremens | 1 |
| Deficient development | 1 |
| Diarrhœa | 9 |
| " chronic | 4 |
| Diphtheria | 11 |
| Dropsy, general | 4 |
| Dropsy of abdomen | 1 |
| Dysentery | 8 |
| " chronic | 1 |
| Embolia | 1 |
| Endo-cervieitis | 1 |
| Enteritis | 13 |
| Entero colitis | 2 |
| Epilepsy | 2 |
| Erysipelas | 3 |
| Exhaustion | 3 |
| Fever, congestive | 1 |
| " puerperal | 5 |
| " remittent | 4 |
| " scarlet | 13 |
| " " malignant | 1 |
| " " and complications | 3 |
| " typhoid | 75 |
| Gangrene | 1 |
| " of bowels | 1 |
| Gastritis | 4 |
| Gastro-enteritis | 5 |
| Hæmatemesis | 1 |
| Hemorrage, internal | 1 |
| " umbilical | 1 |
| Heart, disease of | 5 |
| " inflammation of | 1 |
| " organic disease of, | 1 |
| " vulvular disease of, | 5 |
| Hepatitis | 1 |
| Hip joint, disease of | 1 |
| Hydrocephalus | 9 |
| Icterus | 1 |
| Inanition | 4 |
| Kidneys, Bright's disease of | 2 |
| Laryngitis | 1 |
| Lungs, congestion of | 7 |
| " abscess of | 1 |
| Measles | 1 |
| Metro peritonitis | 1 |
| Meningitis | 7 |
| " cerebro-spinal, | 4 |
| " tubercular | 2 |
| Metritis puerperal | 1 |
| Menth gangrene sore | 1 |
| Neuralgia | 1 |
| " and bronchitis | 1 |
| Old age | 16 |
| Paralysis | 1 |
| Pericarditis | 1 |
| Peritonitis | 5 |
| " traumaiic | 1 |
| " puerperal | 1 |
| Pleurisy | 1 |
| Pharyngitis, gangrenous | 2 |
| Pneumonia | 27 |
| " typhoid | 3 |
| Pyæmia | 1 |
| Scrofula | 1 |
| Small pox | 13 |
| Spina bifida | 2 |
| Stomach, ulceration of | 1 |
| Suicide by cutt.ng throat, | 1 |
| " morphine | 1 |
| Tabes mesenterica | 10 |
| Teething | 4 |
| " and complications | 2 |
| Tetanus | 1 |
| Urine, retention of | 1 |
| Uterus, hemorrage of | 1 |
| Varicella | 1 |
| Vitality deficient | 1 |
| Whooping cough | 4 |
| Total | 516 |
| Premature births | 9 |
| Still births | 36 |
| Total | 45 |

### AGES.

| | | | | | |
|---|---|---|---|---|---|
| Under 1 year | 115 | 70 to 80 | 25 | Married | 153 |
| 1 to 2 | 52 | 80 to 90 | 6 | Single | 363 |
| 2 to 3 | 27 | Unknown | 2 | | — — |
| 3 to 4 | 22 | | — | Total | 516 |
| 4 to 5 | 11 | Total | 516 | | |
| 5 to 10 | 35 | | | | |
| 10 to 20 | 32 | | | | |
| 20 to 30 | 60 | | | Colored | 6 |
| 30 to 40 | 47 | Males | 296 | White | 510 |
| 40 to 50 | 36 | Females | 220 | | — |
| 50 to 60 | 25 | | — | Total | 516 |
| 60 to 70 | 21 | Total | 516 | | |

### MORTALITY BY WARDS.

| Wards. | No. deaths. | Pop'n 1871. | Per ct. 1 d'th in | RECAPITULATION. | |
|---|---|---|---|---|---|
| 1 | ... | 8,103 | ...... | Accidents | 15 |
| 2 | 1 | 13,449 | 13,449 | Barracks | 4 |
| 3 | 26 | 17,934 | 690 | County Hospital | 22 |
| 4 | 8 | 14,022 | 1,556 | Foundling Home | 7 |
| 5 | 16 | 14,991 | 937 | Galena Elevator | 1 |
| 6 | 35 | 22,918 | 655 | Home for Friendless | 2 |
| 7 | 35 | 15,590 | 445 | Mercy Hospital | 10 |
| 8 | 51 | 25,420 | 498 | Newberry School | 1 |
| 9 | 54 | 30,778 | 570 | Protestant Orphan Asylum | 2 |
| 10 | 19 | 17,292 | 910 | Police Station | 1 |
| 11 | 44 | 16,212 | 368 | Small Pox Hospital | 1 |
| 12 | 24 | 15,018 | 626 | St. Luke's Hospital | 2 |
| 13 | 7 | 9,740 | 1,391 | Suicides | 2 |
| 14 | 12 | 9,334 | 778 | | — |
| 15 | 75 | 25,706 | 348 | Total | 516 |
| 16 | 21 | 16,380 | 780 | | |
| 17 | 8 | 18,814 | 2,352 | | |
| 18 | 9 | 18,805 | 2,100 | | |
| 19 | ... | 9,237 | | | |
| 20 | ... | 14,522 | | | |

### NATIVITY.

| | | | | | |
|---|---|---|---|---|---|
| Bohemia | 7 | Germany | 72 | Wales | 1 |
| Canada | 5 | Ireland | 56 | Unknown | 10 |
| Chicago, native | 66 | Norway | 17 | | — |
| " foreign | 164 | Prince Edwa.d's Island | 1 | Total | 516 |
| U. S., other parts | 78 | Poland | 1 | | |
| Denmark | 1 | Scotland | 4 | | |
| England | 16 | Sweden | 17 | Deaths daily | 17.20 |

### COMPARATIVE MORTALITY.

| | 1870. | 1871. | Increase or decrease. |
|---|---|---|---|
| Accidents | 13 | 15 | 2 |
| Apoplexy | 10 | 2 | 8 |
| Asthma | 0 | 2 | 2 |
| Brain diseases | 12 | 18 | 6 |
| Bronchitis | 9 | 5 | 4 |
| Cancer | 6 | 5 | 1 |
| Cholera infantum | 3 | 7 | 5 |
| Consumption | 49 | 32 | 17 |
| Convulsions | 40 | 50 | 10 |
| Croup | 54 | 21 | 33 |
| Debility, general | 7 | 7 | 0 |
| Diarrhœa | 6 | 13 | 7 |
| Diphtheria | 19 | 11 | 8 |
| Dropsy | 4 | 5 | 1 |
| Dysentery | 2 | 9 | 7 |
| Enteritis | 2 | 13 | 11 |
| Fever, congestive | 1 | 1 | 0 |
| " remittent | 3 | 4 | 1 |
| " puerperal | 3 | 5 | 2 |
| " scarlet | 13 | 17 | 4 |
| " typhoid | 35 | 75 | 40 |
| Gastritis | 0 | 9 | 9 |
| Heart diseases | 13 | 16 | 3 |
| Hydrocephalus | 2 | 9 | 7 |

| | 1871. | 1871. | Increase or decrease. |
|---|---|---|---|
| Inanition | 4 | 4 | 0 |
| Lungs, congest'n of | 1 | 7 | 6 |
| Meningitis | 10 | 14 | 4 |
| Old age | 4 | 16 | 12 |
| Peritonitis | 4 | 7 | 3 |
| Pneumonia | 19 | 30 | 11 |
| Small pox | 0 | 13 | 13 |
| Suicide | 4 | 2 | 2 |
| Tabes mesenterica, | 14 | 10 | 4 |
| Teething | 5 | 6 | 1 |
| Whooping cough | 2 | 4 | 2 |
| Total causes of death | 109 | 113 | 4 |
| Under 5 years old | 201 | 227 | 26 |
| 5 to 20 " | 54 | 67 | 13 |
| 20 to 50 " | 123 | 143 | 20 |
| 50 and upwards | 44 | 79 | 35 |
| Males | 231 | 216 | 15 |
| Females | 261 | 219 | 28 |
| Born, Chicago nat., | 58 | 66 | 8 |
| " " for'n | 143 | 164 | 21 |
| " United States, | 64 | 78 | 14 |
| " foreign | 153 | 208 | 55 |
| Total | 422 | 516 | 94 |

Compared with the corresponding month of 1870, there is a decided increase in the number of deaths. There were 2 more by accidents, 2 more by asthma, 6 by brain diseases, 4 by bronchitis, 5 by cholera infantum, 10 by convulsions, 6 by diarrhœa, 7 by dysentery, 11 by enteritis, 2 by puerperal fever, 4 by scarlet fever, 40 by typhoid fever, 9 by gastritis, 3 by heart diseases, 7 by hydrocephalus, 9 by congestion of the lungs, 4 by meningitis, 12 by old age, 3 by peritonitis, 12 by pneumonia, and 13 by small pox; while there were 8 less by apoplexy, 17 by consumption, 33 by croup, 8 by diphtheria, and 4 less by tabes mesenterica.

The most marked feature in the causes of death is the increase by typhoid fever, undoubtedly the direct result of over-crowding. In 1866 there were 249 deaths by this disease, in 1867 198, in 1868 233, in 1869 183, in 1870 222, and so far this year 268. More deaths occurred during this month than any other known in the history of the city from this disease. The only month approaching it is October, 1866, when 51 are reported as dying from this cause, many of them having suffered from cholera. Small pox next attracts attention, as the mortality by this disease has been greater than for the same time in four years. In 1863 there were 113 deaths from this cause, in 1864 383, in 1860 59, and in 1866 9, making a total of 464: while since, in 1867, 123, in 1868 146, 1869 17, 1870 15, and so far this year 27, making a total of 328 for nearly five years. Up to this month the number of deaths by this disease was only 14 for this year. Almost daily for the last six months, emigrants have arrived here suffering from small pox or the infection, and by the exercise of the utmost vigilance by the Board its spread has been prevented, while in other cities it prevailed to a great extent. Since the fire, however, owing to the destruction of the Small Pox Hospital, the general disorganization of everything, the want of knowledge of cases, and the overcrowding of houses, have to some extent prevented ———, vaccination and the other precautions used by the Board, the disease has increased upou us during this month, so that by the end of it there were 74 cases in the city. Although every effort possible has been made to prevent the spread of the disease, under the circumstances in which we are placed, it has not made, comparatively speaking, great headway. There is probably no better instance on record of what can be done in the way of prevention than our experience before and since the fire. In short, we were crippled, and as the result we have an increase.

Diseases of the alimentary canal have also been unusually frequent, no doubt the direct result of change of diet, and exposure. Brain diseases have also been more common, and the deaths by "old age" are much in excess of last pear. A careful analysis of the causes of death satisfies me that fully twenty per cent. of these were either the direct or indirect result of the fire. In fact, I am astonished that it has not been greater. In November, 1867, 373 deaths occurred, in 1868 401, in 1869 489, in 1870 422, and this year 516.

JOHN H. RANCH,
Sanitary Superintendent.

---

MONEY RECEIPTS, SEPTEMBER, OCTOBER, NOVEMBER, 1871.—Dr. Wm. Martin, $3.00; Dr. J. C. Allaben, 3.00; Dr. A. G. Jones, 3.00; Dr. B. F. Rolfe' 5.00; Dr. R. D. Coggswell, 5.00; Dr. H. C. Miner, 3.00; Dr. F. R. Bailey, 5.00; Dr. J. Brewster, 3.00; Dr. J. W. Mott, 3.00; Dr. B. Wilson, 3.00; Dr. J. Lawless, 3 00; Dr. C. F. Falley, 5.00; Dr. Thos. Aiton, 3.00; Dr. M. Mitchell, 1.00; Dr. O. W. Moore, 3.00; Dr. E. O. F. Roller, 3.00; Dr. L. Cuthbert, 1.00; Dr. T. Maxon, 3.oc; Dr. W. L. Pollock, 3.00; Dr. J. Hurley, 3.00; Dr. J. M. Steele, 3.00; Dr. Thos. J. Whitten. 3.00; Dr. O. C. Boffmer, 3.00; Dr. Addison Niles, 3.00; Dr. Perry, 3.00; Dr. W. H. Lyford, 5.00; Dr. O. E. Vandeventer, 5.00; Dr. L. J. Burrows, 6.00; Dr. L. W. Homes, 3.00; Dr. Schofer, 2.50; Dr. D. V. Cole, 6.00; Dr. L. D. Smedley, 6.00; Dr. P. Drumward, 3.00; Dr. McDonald, 3.00; Dr. W. S. Harvey, 3.00; Dr. G. R. Skinner, 5.00; Dr. Jas. M. Barlow, 3.00; Dr. J. H. Mears, 12.00; Dr. E. L. Greenleaf, 9.00; Dr. D. T. Kyner, 3.00; Dr. Jno. M. Woodworih, 4.50; Dr. A. Given, 9.00; Dr. J. J. Hale, 1.50; Dr. J. W. Duncan, 3.00; Dr. O'Donnell, 3.00; Dr. S. E. Mitchell, 6.00.

